# G-patch domain and KOW motifs-containing protein, GPKOW; a nuclear RNA-binding protein regulated by protein kinase A

**DOI:** 10.1186/1750-2187-6-10

**Published:** 2011-08-31

**Authors:** Anne Kristin Aksaas, Anja CV Larsen, Marie Rogne, Ken Rosendal, Anne-Katrine Kvissel, Bjørn Steen Skålhegg

**Affiliations:** 1 Department of Nutrition, University of Oslo, Oslo, Norway; 2 Department of Biochemistry, University of Oslo, Oslo, Norway; 3 Spermatech AS, Oslo, Norway

**Keywords:** RNA processing, phosphorylation, PKA catalytic subunit, GPKOW

## Abstract

**Background:**

Post-transcriptional processing of pre-mRNA takes place in several steps and requires involvement of a number of RNA-binding proteins. How pre-mRNA processing is regulated is in large enigmatic. The catalytic (C) subunit of protein kinase A (PKA) is a serine/threonine kinase, which regulates numerous cellular processes including pre-mRNA splicing. Despite that a significant fraction of the C subunit is found in splicing factor compartments in the nucleus, there are no indications of a direct interaction between RNA and PKA. Based on this we speculate if the specificity of the C subunit in regulating pre-mRNA splicing may be mediated indirectly through other nuclear proteins.

**Results:**

Using yeast two-hybrid screening with the PKA C subunit Cbeta2 as bait, we identified the G-patch domain and KOW motifs-containing protein (GPKOW), also known as the T54 protein or MOS2 homolog, as an interaction partner for Cbeta2. We demonstrate that GPKOW, which contains one G-patch domain and two KOW motifs, is a nuclear RNA-binding protein conserved between species. GPKOW contains two sites that are phosphorylated by PKA in vitro. By RNA immunoprecipitation and site directed mutagenesis of the PKA phosphorylation sites we revealed that GPKOW binds RNA in vivo in a PKA sensitive fashion.

**Conclusion:**

GPKOW is a RNA-binding protein that binds RNA in a PKA regulated fashion. Together with our previous results demonstrating that PKA regulates pre-mRNA splicing, our results suggest that PKA phosphorylation is involved in regulating RNA processing at several steps.

## Background

Post-transcriptional processing of pre-mRNA involves splicing, editing and polyadenylation and starts as soon as pre-mRNAs are produced at their sites of transcription. In addition to small non-coding RNAs (snRNAs) these processes require the activity of RNA-binding proteins (RBPs) [1,2]. The discovery of the heterogeneous nuclear ribonucleoproteins (hnRNP) and other pre-mRNA/mRNA-binding proteins led to the identification of the first amino acid motifs and functional domains that confer binding to RNA [3]. RBPs, which may contain one or multiple RNA-binding domains, are important for RNA metabolism and are involved in RNA stability, splicing, transport, cellular localization, translation and decay [4]. The posttranscriptional RNA regulon model suggests that functionally related mRNAs are co-regulated by RBPs and snRNAs. This allows for a smooth and coordinated regulation of mRNAs and protein synthesis according to specific tasks [5].

Little is known about how signal transduction pathways may be involved in regulating post-transcriptional processing of pre-mRNA. For alternative splicing, it is established that reversible phosphorylation conducts some of these signals to the spliceosome [6]. We have recently demonstrated that the C subunit of PKA is located to splicing factor compartments (SFC) where it regulates alternative pre-mRNA splicing in a cAMP-independent manner [7]. PKA, which is a holoenzyme consisting of a regulatory (R) subunit dimer and two C subunits, is expressed in all human cells and tissues examined. There are four genes encoding the R subunits RIα, RIβ, RIIα, RIIβ, and the C subunits Cα, Cβ, Cγ and PrKX, respectively. Upon activation, the R subunit dimer binds four molecules of cAMP leading to the dissociation of the C subunits and phosphorylation of relevant substrates in the cytoplasm and the nucleus [8]. Specificity of PKA signalling in the cell is thought to partly be maintained through targeting of the R subunits to subcellular organelles through A-kinase anchoring proteins (AKAPs) [9]. How activity and specificity of the C subunits are regulated after dissociation from the R subunits and the corresponding AKAP, in addition to the assumption that the R subunits are absent from the nucleus is not well understood. It is however believed that temporal and spatial regulation may involve both phosphorylation and association of the C subunit with different target proteins. It is well documented that the C subunit phosphorylates and regulates the activity of a vast number of proteins including nuclear cAMP response element-binding proteins (CREBs) [10]. Moreover, C subunit binding proteins have also been identified, and include the cytoplasmic proteins IκB [11], Caveolin-1 [12] and p75 neurotrophin receptor (P75^NTR^) [13] as well as the nuclear proteins protein kinase inhibitor (PKI) [14], homologous to AKAP95 (HA95) [15], A-kinase-interacting protein (AKIP) [16] and heat shock factor 1 [17].

Using yeast two-hybrid screening with the PKA Cβ2 subunit as bait we identified GPKOW, also known as MOS2 homolog or T54 protein, as an interaction partner for Cβ2. There is currently little knowledge about GPKOW. We demonstrate that it is a nuclear protein that binds RNA in a PKA-regulated fashion *in vivo; *indicating that PKA may be involved in regulating multiple steps in post-transcriptional processing of pre-mRNAs.

## Materials and methods

### Yeast two-hybrid screening

Yeast two-hybrid screen using the PKA C subunit Cβ2 as bait was carried out by Dualsystems Biotech AG, Zurich, Switzerland. The bait construct was made by subcloning of a cDNA encoding complete Cβ2 into the pLexA-DIR (Dualsystems Biotech AG, Zurich, Switzerland). The bait construct was transformed into the strain DSY-1 (MATa his3200 trp1-901 leu2-3,112 ade2 LYS2::(lexAop)4-HIS3 URA3::(lexAop)8-lacZ GAL4) using standard procedures [18]. Correct expression of the bait was verified by western blotting of cell extracts using a mouse monoclonal antibody directed against the LexA domain (Santa Cruz Biotechnology, Santa Cruz, California, USA). The absence of self-activation was verified by co-transformation of the bait together with a control prey and selection on minimal medium lacking the amino acids tryptophan, leucine and histidine (selective medium). For the yeast two-hybrid screen, the bait was co-transformed together with a human peripheral blood cDNA library into DSY-1. Six point four million transformants were screened yielding 22 transformants that grew on selective medium. Positive transformants were tested for β-galactosidase activity using a filter assay [19]. Twenty one of the 22 initial positives showed β-galactosidase activity. Library plasmids were isolated from positive clones and assayed in a bait dependency test with pLexAdir with Cβ2 as bait plasmid and pLexA-p53 and pLexA-laminC plasmids as control bait encoding a LexA-laminC fusion using a mating strategy [20]. Seventeen of the 21 positives showed β-galactosidase activity when co-expressed with the bait but not when co-expressed with the control bait, and were considered to be bait-dependent interactors. The identity of the interactors was determined by sequencing.

### Expression plasmids

DNA for the native PKA subunits Cα1, Cβ1, Cβ2, Cβ4 and RIα, in addition to the C-terminal V5-tagged Cβ2, was PCR amplified from cDNA from NT2-N cells. PCR products were cloned into expression plasmids using the pENTR/D-TOPO Cloning Kit (Invitrogen 45-0218) and the mammalian expression vector pEF-DEST51 (Invitrogen 12285-011) by the LR Clonase reaction (Invitrogen 11791-019) according to the manufacturer. Full-length GPKOW was PCR-amplified from Jurkat cell cDNA using the following primers: upper GPKOW F TOPO (5'CACCATGGCTGACTCCAAAGAGGG3') and GPKOW R (5'GTCATCATCTGTGTCACTAGG3') for constructs without stop codon or GPKOW stop R (5'TCAGTCATCATCTGTGTCACTAG3') for constructs with stop codon. The GPKOW PCR products were cloned into expression plasmids using the pENTR/D-TOPO Cloning Kit (Invitrogen 45-0218) and the mammalian expression vector pEF-DEST51 (Invitrogen 12285-011) by the LR Clonase reaction (Invitrogen 11791-019) according to the manufacturer. The resulting plasmids expressed full-length GPKOW with or without a C-terminal V5-tag. The plasmids for prokaryote expression were made by PCR-amplification of Jurkat cDNA using following primers: GPKOW Nde1 F (5'TACGCATATGGCTGACTCCAAAGAGGGTGT3') and GPKOW C-His BamHI R (5'TACGGGATCCTCAGTGATGGTGATGGTGATGGTCATCATCTGTGTCACTAGG3' to introduce Nde1 and BamHI restriction sites and a C-terminal 6 × His-tag. Digested PCR products were cloned into the pET24b vector (Novagen 69750). The QuickChange mutagenesis kit (Stratagene 200524-5) was used to generate GPKOW full-length with mutations in the PKA phosphorylation sites with primers GPKOWfos176U23 (5'CACTCGCACGGCCGCACGGAGGC3') and GPKOWfos176L23 (5'GCCTCCGTGCGGCCGTGCGAGTG3') to change serine to alanine at position 27. The primers GPKOWfos2940U26 (5'CTCATCACGGAAGGCCCTCTGGAATC3') and GPKOWfos2940L26 (5'GATTCCAGAGGGCCTTCCGTGATGAG3') were used to changed threonine to alanine at position 316. All primers were bought from Sigma-Genosys and all expression plasmids were sequenced.

### Cell culture

293T cells were maintained at 37°C in humidified air with 5% CO_2 _in RPMI 1640 (Sigma-Aldrich R0883) supplemented with 10% foetal bovine serum (Sigma-Aldrich F7524), 1% non-essential amino acids (GibcoBRL 11140-035), 1% L-glutamine (Sigma-Aldrich G7513), 1% sodium pyruvat (GibcoBRL 11360-039) and 1 × penicillin-streptomycin solution (Sigma-Aldrich P4458). The 293T cells were transfected with Lipofectamine 2000 (Invitrogen 11668-019) according to the manufacturer's protocol. U2OS cells were grown in DMEM (Sigma-Aldrich D6546) supplemented with 10% foetal bovine serum (Sigma-Aldrich F7524), 1% L-glutamine (Sigma-Aldrich G7513) and 1 × penicillin-streptomycin solution (Sigma-Aldrich P4458). U2OS cells were transfected with Fugene6 (Roche 11814443001) according to the manufacturer's protocol.

### Co-immunoprecipitation

Four wells (35 mm) with transfected 293T cells were washed in PBS and lysed by sonication in immunoprecipitation buffer (150 mM NaCl, 50 mM Tris pH 7.5, 0.5% Triton X-100, 1 × protease inhibitor cocktail (Sigma P8340), 1 mM PMSF (Sigma P7626) and 1 mM Na_3_VO_4 _(Sigma S6508)). The lysates were cleared by centrifugation and adjusted to equal protein concentration for each experiment (between 1.5 - 3.4 μg protein/μl) by the Bradford method (BioRad 500-0006). The lysates were precleared with Dynabeads Protein G (Invitrogen 100-04D, 1:10) before input samples were collected (20 μl) and the remainder of the lysates were added one of the following antibodies; rabbit IgG (same amount μg as the positive immunoprecipitation antibody, Sigma I5006), anti-GPKOW (1.6 μg, Atlas Antibodies HPA001894), anti-PKA RIα serum (6 μl)[21] or anti-PKA Cβ2 (12.8 μg, clone SNO103 from rabbit serum)[22]. The mixture of cell lysate and antibody were incubated at 4°C rotating over night before Dynabeads Protein G (1:10) were added the next day and incubated for another hour. The samples were washed with immunoprecipitation buffer, added SDS loading dye and boiled for 5 minutes before immune blotting. For samples treated with cAMP, the magnetic beads were after washing incubated 5 min with buffer with or without 1 mM 8-CPT (Sigma C3913) and both pellet and the supernatant were analyzed by immunoblotting.

### Dephosphorylation assay

The procedure is according to the manufacturer's recommendation (Sigma product P0114)[23]. Two wells with 293T cells were washed in PBS and lysed by sonication in immunoprecipitation buffer (150 mM NaCl, 50 mM Tris pH 7.5, 0.5% Triton X-100, 1 × protease inhibitor cocktail (Sigma P8340), 1 mM PMSF (Sigma P7626) and 1 mM Na_3_VO_4 _(Sigma S6508)). The lysates were cleared by centrifugation and adjusted to equal protein concentration (between 2.5 - 3.0 μg protein/μl) by the Bradford method (BioRad 500-0006). The lysates were precleared with Dynabeads Protein G (Invitrogen 100-04D, 1:10) and incubated over night with anti-GPKOW (0.8 μg, Atlas Antibodies HPA001894). Dynabeads Protein G (1:10) were added the next day and incubated for another hour. The samples were washed with immunoprecipitation buffer. Magnetic beads were added 100 units of alkaline phosphatase (Sigma P0114) in 5 mM Tris pH 7.9, 10 mM NaCl, 1 mM MgCl_2 _and 0.1 mM DTT for 30 minutes at 30°C or left untreated on ice. The samples were added SDS loading dye and boiled for 5 minutes before immune blotting.

### Immunoblotting

If not otherwise stated, the cells were washed in ice cold PBS and lysed by sonication in lysis buffer (150 mM NaCl, 50 mM Tris pH 7.5, 0.5% Triton-X100, 1 mM Na_3_VO_4_, 1 mM PMSF and 1 × Protease Inhibitor Cocktail (Sigma-Aldrich P8340)). Lysates were cleared by centrifugation at 16.000 × g for 20 minutes at 4°C. Equal amounts of protein were separated by SDS-PAGE and transferred to a PVDF membrane. The membrane was blocked by drying and then rehydrated in methanol before incubation with primary antibodies; anti-GPKOW 0287 (1:250 dilution, Atlas Antibodies HPA000287), anti-GPKOW1894 (1:500 dilution, Atlas Antibodies HPA001894), anti-GPKOWB01 (1:1000 dilution, Abnova H00027238-B01), purified mouse anti-PKAc (1:250 dilution, BD Transduction Laboratories 610981), anti-PKA RIα (1:250)[21] or anti-V5 (1:2000, Invitrogen R960-25) for one hour at room temperature (RT). The membrane was washed, before incubating with the secondary antibodies; HRP conjugated goat anti-mouse (1:2000 dilution, ICN Biomed 55563) or HRP conjugated goat anti-rabbit (1:2000 dilution, ICN Biomed 55689) for one hour at RT. After washing, immunoreactive proteins were visualized using the ECL detection system and developed using Hyperfilm or the Syngene G:BOX imaging system.

### Immunofluorescence

The procedure was performed as previously described [7]. The following antibodies were used; primary antibodies: anti-GPKOW1894 (1:100), anti-V5 (1:2000) and a rabbit polyclonal antibody against a peptide of human B-type lamins (1:1000)[24] and secondary antibodies: Alexa Fluor 488 goat anti-rabbit IgG (1:400 dilution, Invitrogen A11008) and Alexa Fluor 594 goat anti-mouse IgG (1:400, Invitrogen A11005). To check the specificity of the GPKOW-antibody, it was pre-incubated with its blocking peptide (Atlas Antibodies, PrEST antigen for GPKOW HPA001894).

### RNA immunoprecipitation

The method is according to Rogne et al [25] with some minor modifications. For the endogenous samples, 293T cells were grown confluent in 150 cm^2 ^petri dishes (sufficient for 2 samples), while four 10 cm^2 ^wells were used for the transiently transfected 293T cells. Culture medium was replaced by PBS before UV irradiation (254 nm, 200 mJ/cm^2^, twice). Cells were harvested, washed in PBS and lysed in 300 μl IPB buffer (10 mM HEPES pH 7.5, 2 mM EDTA, 10 mM KCl, 1% TX-100, 1 mM PMSF, 1 × Protease Inhibitor Cocktail (Sigma-Aldrich P8340)) with 150 mM NaCl pr sample before sonication. Lysates were cleared by centrifugation at 15,000 × g for 20 minutes at 4°C before addition of 0.048 U/μl (final concentration) of T1 RNase (Fermentas EN0541) and incubation for 8 minutes at 37°C with 1000 rpm agitation. The lysates were adjusted to equal protein concentrations and precleared with Dynabeads Protein G (Invitrogen 100.04D) for 30 minutes at 4°C rotating. Antibody (0.8 μg anti-GPKOW 1894 or 0.8 μg rabbit IgG, Sigma 1-5006) and beads were incubated on a rotator for 2 hours at 4°C followed by three washes in IPB buffer. Supernatants were incubated with 30 μl conjugated beads for 2 hours at 4°C rotating. The immunoprecipitated samples were washed 4 times by alternating between 1 ml RIPA (1 × PBS, 0.05% SDS, 0.1% deoxycholate, 1% NP-40, 1 × Protease Inhibitor Cocktail (Sigma-Aldrich P8340)) and RIPA 1000 (1 × PBS, 1 mM EDTA, 0.05% SDS, 0.1% deoxycholate, 1 M NaCl, 1% NP-40, 1 × Protease Inhibitor Cocktail (Sigma-Aldrich P8340)). Immunoprecipitated samples were dephosphorylated (0.038 U/μl calf intestinal phosphatase for 10 min, Roche 10713023001) and washed three times with Buffer C (50 mM Tris HCl, pH 7,4, 10 mM MgCl2, 0,5% NP-40, 1 × protease inhibitor cocktail (Sigma-Aldrich P8340)), followed by labeling with γ-^32^P ATP (2.1 μCi, Nerliens NEG502H) by polynucleotide kinase (PNK, 0.5 U/μl NEB M0201L) phosphorylation and further washed three times with Buffer C. All samples were resolved in FA dye and separated by electrophoresis on a denaturing (7 M urea) 6% polyacrylamide (PAA) gel. Incorporated phosphate was visualized by autoradiography. Unsaturated images from the Typhoon 9410 phosphoimager (GE Healthcare Life Sciences) were quantified using the histogram function in Adobe Photoshop. The lanes were manually defined with identical frames. The statistical analysis was performed with paired students T-test in Prism GraphPad.

### Protein purification

Plasmids were transformed into Rosetta 2(DE)pLys bacteria and grown on LB agar plates. 10-20 colonies were picked and grown in 50 ml LB-medium over night at 37°C. The bacteria were pelleted and resuspended in 500 ml TB-medium and grown to optic density between 0.5-0.7 at 600 nm at 37°C and 150 rpm, before protein expression was induced by 0.25 mM IPTG and further grown for 4 hours at 25°C at 150 rpm. The bacteria were lysed in Buffer A (50 mM sodium dihydrogen phosphate pH 7.5, 0.2 M NaCl, 5 mM Imidazole, 1 × EDTA free protease inhibitor cocktail (Roche 04693132001) and 0.4 mM betamercaptoetanol) and purified on a crude HisTrap column (GE Healthcare 11-0004-58) using Buffer A and Buffer B (50 mM sodium dihydrogen phosphate pH 7.5, 0.2 M NaCl, 500 mM Imidazole, 1 × EDTA free protease inhibitor cocktail (Roche 04693132001) and 0.4 mM betamercaptoetanol). Fractions with the highest optical density at 280 nm were pooled, dialyzed to Buffer C (20 mM Tris-HCl, pH 8) and further purified by ion exchange chromatography (Resource Q, GE Healthcare 520348) at pH 8 with buffer C and buffer D (20 mM Tris-HCl pH 8, 1 M NaCl). Fractionated proteins were dialyzed to GPKOW storage buffer (10 mM Tris-HCl pH 7.5, 100 mM NaCl, 1 × protease inhibitor cocktail and 0.4 mM betamercaptoetanol) and stored at 4°C.

### *In vitro *phosphorylation assay

Purified GPKOW proteins were *in vitro *phosphorylated as previously described [7]. As a negative control for phosphorylation heat-inactivated PKA Cα1 was used. Unsaturated images from Syngene G-box and Typhoon 9400 phosphoimager were quantified by Genetools (Syngene) and the statistical analysis was performed with paired students T-test in GraphPad Prism. The values from the phospho blot were adjusted according to the amount of protein detected on the Coomassie Brilliant Blue (CBB)-stained gel.

## Results

**Figure 1:**
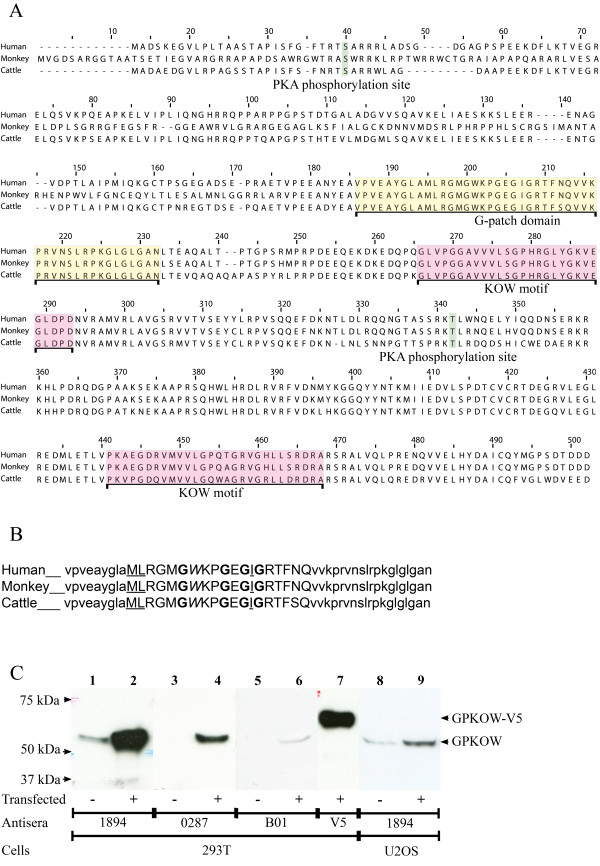
**Sequence, domains and expression of GPKOW**. **A**. Sequence alignment of GPKOW from human (NCBI NP_056513.2, upper line), monkey (NCBI XP_001105674, middle line) and cattle (NCBI XP_616889, lower line). The G-patch domain is indicated in yellow and the two KOW motifs are indicated in red. Two potential PKA phosphorylation sites, S27 and T316 (according to human GPKOW), are indicated in green. **B**. The G-patch domain of GPKOW as defined in the ELM database. Upper case letters illustrate the G-patch consensus sequence, where bold letters are glycines, underscored letters are hydrophobic residues and aromatic amino acids are marked in italic. **C**. 293T cells were left untreated (lanes 1, 3 and 5) or transfected with full-length GPKOW (lanes 2, 4 and 6) or GPKOW with a C-terminal V5-tag (lane 7). U2OS cells were left untreated (lane 8) or transfected with full-length GPKOW (lane 9). Twenty hours post transfection the cells were harvested and lysed. Eighteen μg (lanes 1-4 and 7), 36 μg (lane 5-6) or 46 μg (lanes 8-9) of protein was loaded per lane, separated by SDS-PAGE (10% gels) and analyzed by immunoblotting using three different GPKOW antibodies, 1894 (lanes 1-2 and 8-9), 0287 (lanes 3-4) and B01 (lanes 5-6) or V5 antibody (lane 7). One representative immunoblot from each experiment is presented.

To identify putative novel interaction partners of the lymphoid tissue-enriched PKA C subunit Cβ2, a human peripheral blood cDNA library was screened using a LexA based yeast two-hybrid system by Dual Systems Biotech [26] (Additional files 1 and 2). Twelve bait dependent clones were identified as PKA RIα and validated the two-hybrid screen. Five of seventeen bait dependent clones corresponded to the full-length version of GPKOW according to the NCBI Gene bank database accession number BC003148 (Figure 1A). In addition we identified the sequence on two non-bait dependent clones (β2 microglobulin and eukaryotic translation elongation factor 1 alpha 1). GPKOW, which was discovered during the mapping of chromosome Xp11.23-22 [27], is also referred to as Protein T54 or MOS2 homolog and got its name from the two domains; G-patch and KOW (Figure 1A, yellow and red boxes respectively). As defined by the ELM database, the two KOW motifs of human GPKOW span the amino acids 240 through 267 and 415 through 442. The KOW regions detected here are in agreement with the general definition of related domains identified in 1883 proteins (Pfam database). The KOW motif should encompass approximately 27 amino acids, in which a glycine at position 11 (position 13 in the KOW motifs in GPKOW) should be conserved and surrounded by alternating blocks of polar and nonpolar amino acids [28]. One domain of GPKOW (Figure 1B) is consistent with a G-patch domain of approximately 48 amino acids that has been identified in 775 proteins (Pfam database). Its consensus sequence consists of 6 highly conserved glycines according to the following sequence; hhx(3)Gax(2)GxGhGx(4)G, where the "h" represents bulky hydrophobic residues, the "a" represents an aromatic residue and the "x(n)" represents any residues [29]. In GPKOW, the same pattern was identified except for the fifth glycine which is substituted with a glutamine. A sixth conserved glycine is not included in the consensus sequence, but according to Aravind and Koonin [29] it is located as the 15th amino acid after the domain end. GPKOW has a conserved glycine in this position.

Full-length GPKOW was cloned into the Gateway system for eukaryote expression with and without a C-terminal V5 tag. To identify expressed GPKOW, we applied three polyclonal GPKOW antibodies and a V5-antibody. This demonstrated that all antisera reacted against the expressed proteins of the expected theoretical sizes of 52.5 kDa (full-length) and 57.5 kDa (V5-tagged GPKOW) (Figure 1C). Anti-GPKOW1894 revealed the best titer and was reactive against both expressed and endogenous protein and was therefore primarily used.

**Figure 2:**
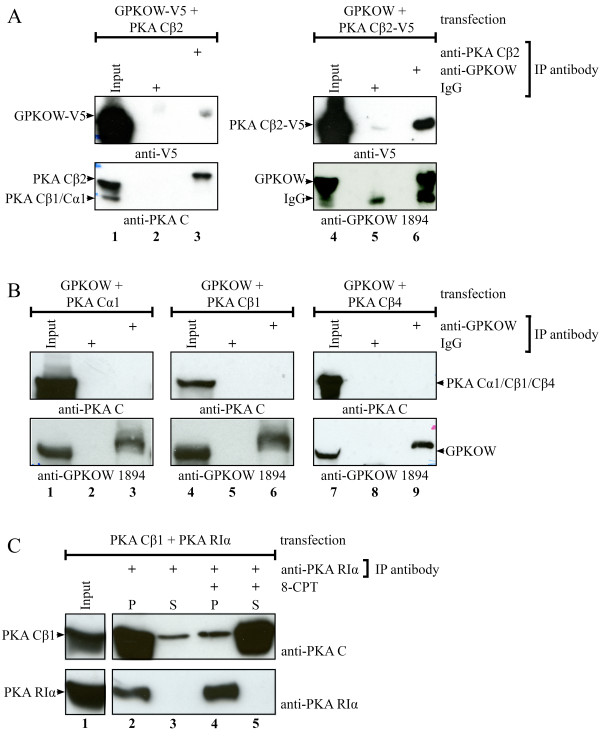
**Co-immunoprecipitations of GPKOW and PKA Cβ2**. **A**. 293T cells were transfected with native PKA Cβ2 and GPKOW with a C-terminal V5-tag (lanes 1-3) or with native GPKOW and PKA Cβ2 with a C-terminal V5-tag (lanes 4-6). Twenty hours post transfection the cells were harvested and lysed. The lysates were adjusted to equal protein concentration for each experiment and precleared with magnetic beads before input samples were collected (lanes 1 and 4). The lysates were subjected to immunoprecipitations using anti-PKA Cβ2 (lane 3), anti-GPKOW (lane 6) or rabbit IgG (lanes 2 and 5) and magnetic beads. All samples were analyzed by SDS-PAGE and immunoblotting using anti-PKA C (lower panel lanes 1-3), anti-GPKOW (lower panel lanes 4-6) or anti-V5 (upper panel). One representative experiment clearly showing interaction is presented. **B**. 293T cells were co-transfected with native GPKOW and PKA Cα1 (lanes 1-3), PKA Cβ1 (lanes 4-6) or PKA Cβ4 (lanes 7-9) and treated as described in A. Input samples (lane1, 4 and 7) and immunoprecipitations with anti-GPKOW (lane 3, 6 and 9) or rabbit IgG (lanes 2, 5 and 8) were immunoblotted with anti-GPKOW (lower panel) or anti-PKA C (upper panel). One representative experiment is presented. **C**. 293T cells were co-transfected with PKA Cβ1 and PKA RIα, treated as described in A and immunoprecipitated with anti-PKA RIα (lanes 2-5). One pellet were treated with the cAMP analog, 8-CPT (lanes 4-5), and all pellets (P) and supernatants (S) were analyzed by SDS-PAGE and immunoblotting using anti-PKA C (upper panel) or anti-PKA RIα (lower panel). One representative experiment is shown.

To explore the binding capability of GPKOW and PKA Cβ2, we co-expressed GPKOW (wild-type (wt) or V5- tagged) and Cβ2 (wt or V5-tagged) in 293T cells and performed cross-immunoprecipitations (Figure 2A). This demonstrated that anti-Cβ2 pulled down GPKOW-V5 (Figure 2A lane 3) and *vice versa *(Figure 2A lane 6). It should be noted that the interaction was inconsistent since approximately 30% of the experiments demonstrated a clear cut interaction. To control for specificity, we co-transfected GPKOW with the PKA subunits Cα1, Cβ1 and Cβ4. In none of these experiments we observed co-immunoprecipitations (Figure 2B lanes 3, 6 and 9). This suggested that GPKOW has a higher preference for interacting with Cβ2 compared to other well characterized PKA C subunits. Despite this, due to the inconsistency observed in the co-immunoprecipitations between GPKOW and Cβ2, the experimental conditions of the immunoprecipitations were tested by co-transfecting Cβ1 with its main endogenous receptor, PKA RIα. Following immunoprecipitation with anti-RIα and incubation in the presence (+) or absence (-) of 8-CPT-cAMP we could demonstrate that anti-RIα immunoprecipitated Cβ1 in a cAMP-dependent fashion, implying satisfactory IP-conditions (Figure 2C). We next investigated if the interaction between Cβ2 and GPKOW was influenced by altered levels of endogenous R after transfection with Cβ2, a situation that may have buffered the availability of expressed Cβ2. After transfecting cells with GPKOW and Cβ2 we stimulated with three different agonists promoting dissociation of the PKA holoenzyme and release of the C subunits. These included the PKA activator 8-CPT-cAMP, the adenylyl cyclase activator forskolin and prostaglandin E2 (PGE2) which is an agonist for the receptor for E-series of prostaglandins [30,31]. None of these treatments improved the rate of interaction (results not shown). Based on these experiments we conclude that the apparent interaction between GPKOW and Cβ2 is weak or highly volatile.

**Figure 3:**
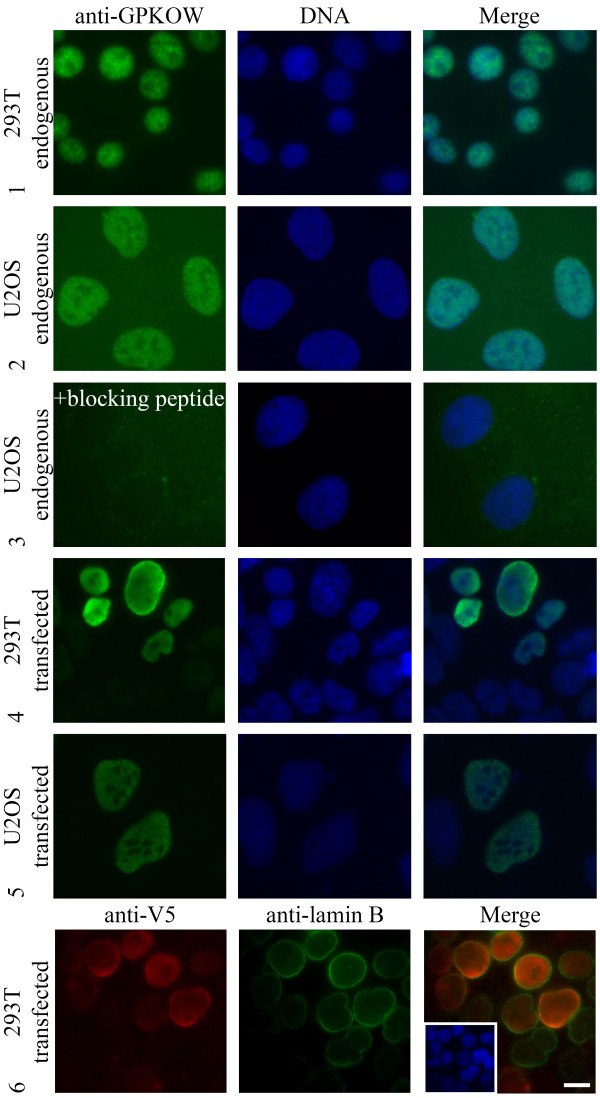
**Localization of GPKOW**. Immunofluorescence analysis of paraformaldehyde fixated 293T (rows 1, 4 and 6) and U2OS cells (rows 2, 3 and 5) stained with anti-GPKOW 1894 (rows 1-5, green), anti-V5 (row 6, red) or anti-lamin B (row 6, green). In the fourth row the antibody was preincubated with antigen (+ blocking peptide) before incubation. The three lower rows show anti-GPKOW and anti-V5 staining of transfected full-length (rows 4-5) or V5-tagged (row 6) GPKOW. Nuclear DNA was visualized with Hoechst (rows 1-6, blue). Merged photos to the right. Scale bar, 10 μM.

Subcellular localization of GPKOW was assessed by immunofluorescence analysis and demonstrated a punctuate distribution in the nucleus. GPKOW appeared to be absent in the cytoplasm and nucleoli of 293T- and U2OS cells (Figure 3, rows 1-2). To ensure specificity of the antibody, we preincubated anti-GPKOW1894 with its antigen (denoted as blocking peptide). This led to complete abolishment of nuclear staining, further substantiating that the immunoreactive protein represents endogenous GPKOW located in the nucleus (Figure 3, row 3). To further confirm localization, we transfected 293T- and U2OS cells with full-length or V5-tagged GPKOW and performed immunofluorescence staining with anti-GPKOW1894 or anti-V5. This revealed that transfected GPKOW was located to the nucleus and appeared to be absent from the cytoplasm and nucleoli (Figure 3, rows 4-6). It should however be mentioned that over-expressed full-length and V5-tagged GPKOW in 293T cells tended to accumulate at the inner surface of the nuclear membrane, as determined by co-staining of anti-V5 with the nuclear membrane marker anti-lamin B (Figure 3, row 6). We did not pursue this phenomenon further since it was considered a result of over-expression and not specific localization. This conclusion was supported in that over-expressed GPKOW in U2OS was distributed in the nucleus in the same manner as the endogenous protein (Figure 3, row 5).

**Figure 4:**
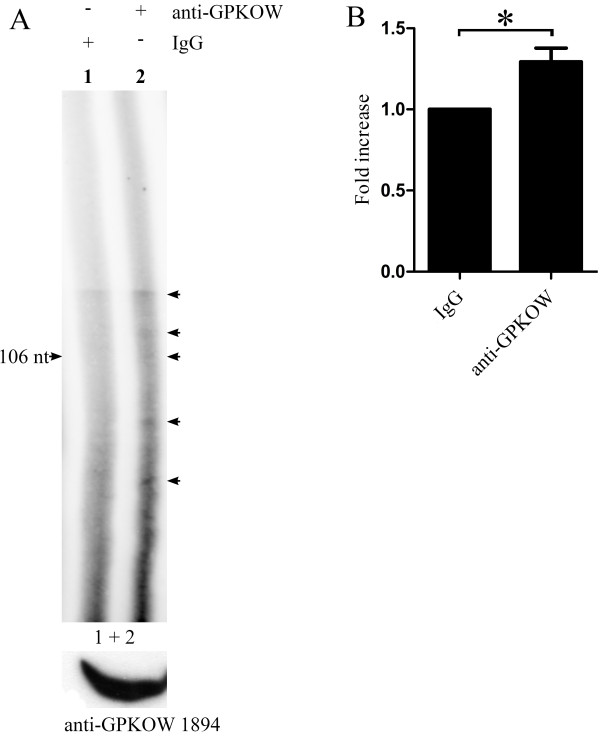
**GPKOW binds RNA *in vivo***. **A**. 293T cells were harvested and exposed to UV irradiation (254 nm/200 mJ). The cells were lysed and treated with T1 RNase (0.048 U/μl) for 8 min followed by immunoprecipitation with Dynabeads conjugated with anti-GPKOW1894 (+ anti-GPKOW, lane 2) or rabbit IgG (+ IgG, lane 1). Immunoprecipitated samples were dephosphorylated with calf intestinal phosphatase (0.038 U/μl). The RNA was labeled with γ^32^-P ATP by PNK (0.5 U/μl) phosphorylation. All samples were separated on a denaturating (7 M urea) 6% PAA gel and subjected to autoradiography. The arrows indicate specific RNA species. The lower panel shows immunoreactive GPKOW from the cell lysate used (one representative lane is shown). **B**. Unsaturated images from the Typhoon 9410 phosphoimager (GE Healthcare Life Sciences) were quantified using the histogram function in Adobe Photoshop. The lanes were manually defined with identical frames. The statistical analysis was performed with paired students T-test in GraphPad Prism. The asterisk indicates a significant difference between the columns with a p-value < 0.05 (n = 4).

It is speculated that the G-patch domain of GPKOW forms two α-helixes that are capable of binding single-stranded RNA and DNA as well as proteins [29,32-34]. In addition, it has been demonstrated that KOW motifs contain alternating hydrophilic and hydrophobic residues that form two β-strands, which have non-overlapping binding sites for both nucleic acids and amino acids. The KOW motif is a close structural homolog of the Tudor protein-protein interaction motif [35-37]. Together this implies that GPKOW may be a nuclear nucleic acid binding protein. To investigate this *in vivo *we performed RNA immunoprecipitation. The sample immunoprecipitated with anti-GPKOW1894 (Figure 4A, lane 2) contained significantly more (p < 0.05, n = 4) radiolabelled nucleotides than the sample immunoprecipitated with rabbit IgG (Figure 4A, lane 1 and Figure 4B), concluding that GPKOW is a RNA-binding protein.

**Figure 5:**
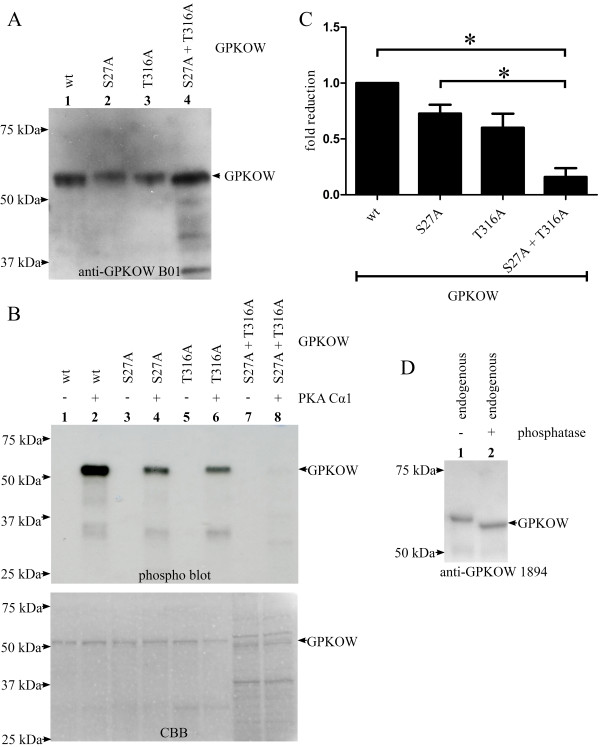
**PKA phosphorylates GPKOW at S27 and T316 *in vitro***. **A**. Recombinant GPKOW proteins were expressed in bacteria and purified on a HisTrap column followed by an ion exchange column. GPKOW wt (lane 1), GPKOW S27A (lane 2), GPKOW T316A (lane 3) and GPKOW S27A+T316A (lane 4) were all recognized by anti-GPKOW B01 after separation on SDS-PAGE and immunoblotting. One representative immunoblot is shown. **B**. Purified GPKOW wt (lanes 1 and 2), single- (lanes 3-6) or double-mutated GPKOW (lanes 7 and 8) were incubated with active (+) or heat inactivated (-) PKA Cα1 (7.4 ng) and γ-^32^P-ATP in a reaction buffer. The samples were analyzed by SDS-PAGE and autoradiography. The CBB staining in the lower panel shows the amount of the different proteins. **C**. Unsaturated images from Syngene G-box and Typhoon 9400 phosphoimager were quantified by Genetools (Syngene) and the statistical analysis was performed with paired students T-test in GraphPad Prism. The asterisk indicates a significant difference between the columns with a p-value < 0.01 (n = 3). **D**. 293T cells were harvested and lysed. The lysates were adjusted to equal protein concentration, precleared with magnetic beads before immunoprecipitation using anti-GPKOW (lane 1-2) and magnetic beads. Sample 2 was treated with alkaline phosphatase for 30 minutes before both samples were analyzed by SDS-PAGE and immunoblotting using anti-GPKOW. One representative experiment is shown (n = 3).

We noticed two potential PKA phosphorylation sites in human GPKOW, at serine 27 (S27) and threonine 316 (T316), which also were present in GPKOW of monkey and cattle (Figure 1A, green box, ELM database)[38]. Based on this observation, we characterized a potential role of PKA in regulating GPKOW function through phosphorylation. First we tested if PKA was capable of phosphorylating GPKOW *in vitro *and further if phosphorylation was involved in regulating GPKOWs RNA-binding properties. Full-length GPKOW wt and GPKOW containing mutations from serine to alanine at position 27 (S27A) and/or threonine to alanine at position 316 (T316A) were expressed with a C-terminal 6 × His-tags in bacteria. Purified proteins were analyzed by immunoblotting using anti-GPKOWB01 and CBB staining, showing that wt and mutated proteins all migrated with an apparent molecular mass of 52.5 kDa (Figure 5A and 5B lower panel). We next tested if the purified GPKOW proteins could be phosphorylated by PKA *in vitro*. This demonstrated that active Cα1 incorporated radiolabelled phosphate in GPKOW (Figure 5B, upper panel, lane 2), whereas phosphorylation was abolished when Cα1 was heat inactivated (Figure 5B, upper panel, lane 1). In the case of the single mutations of S27A and T316A, respectively (Figure 5B, upper panel, lanes 3-6), phosphorylation was decreased compared to wt. For the double mutated GPKOW (Figure 5B, upper panel, lanes 7 and 8 and Figure 5C) phosphorylation was significantly reduced (p- < 0.01, n = 3). It appeared that both S27 and T316 are phosphorylated by PKA Cα1 *in vitro*. In these experiments the amount of the different proteins was determined by immunoblot analysis (Figure 5A) and CBB staining (Figure 5B, lower panel). Longer exposure of the gel revealed a faint bond in the double mutant sample which corresponded to the lower of the two bands at approximately 50 kDa seen with the CBB staining. To investigate if GPKOW is a phospho-protein *in vivo *we conducted a phosphatase treatment experiment. Endogenous GPKOW from 293T cells was immunoprecipitated, incubated with alkaline phosphatase and subjected to immunoblotting. This resulted in a marked reduction in size of the phosphatase treated GPKOW (Figure 5D), supporting that GPKOW also is phosphorylated *in vivo*.

**Figure 6:**
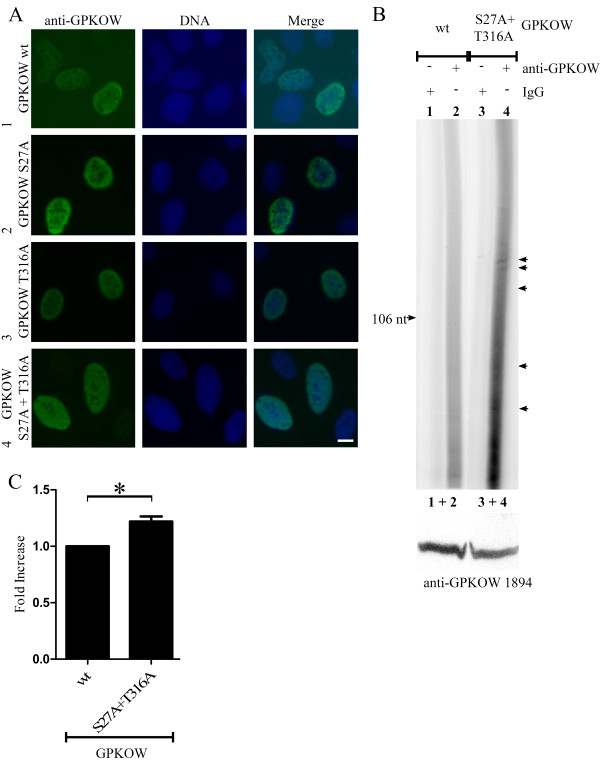
**GPKOWs ability to bind RNA is sensitive to mutations of its PKA phosphorylation sites**. **A**. U2OS cells were transfected with GPKOW wt (row 1), GPKOW S27A (row 2), GPKOW T316A (row 3) or GPKOW S27A+T316A (row 4). Cells were fixated with paraformaldehyde 20 hours later and subjected to immunofluorescence analysis using anti-GPKOW1894 (left, green). Nuclear DNA was visualized with Hoechst (middle, blue). Merged photos to the right. Scale bar 10 μM **B**. 293T cells transfected with GPKOW wt (lanes 1 and 2) or GPKOW S27A + T316A (lanes 3 and 4) were harvested and exposed to UV irradiation at 254 nm (200 mJ). The cells were lysed and immunoprecipitated with Dynabeads conjugated with either rabbit IgG (+ IgG, lanes 1 and 3) or anti-GPKOW1894 (+ anti-GPKOW, lanes 2 and 4). Immunoprecipitated samples were dephosphorylated with calf intestinal phosphatase (0.038 U/μl) followed by labeling of RNA with PNK (0.5 U/μl) phosphorylation. The samples were separated in a denaturating (7 M urea) 6% polyacrylamide gel and subjected to autoradiography. The arrows indicate specific RNA species. The lower panel shows immunoreactive GPKOW from the cell lysate used (one representative lane is shown). n = 4 **C**. Unsaturated images from the Typhoon 9410 phosphoimager (GE Healthcare Life Sciences) were quantified using the histogram function in Adobe Photoshop. The lanes were manually defined with identical frames. The statistical analysis was performed with paired students T-test in GraphPad Prism. The asterisk indicates a significant difference between the columns with a p-value < 0.01 (n = 4).

Next, we speculated if PKA-dependent phosphorylation influenced the localization of GPKOW, as has been demonstrated for other proteins [13,39,40]. Comparison of the expression of wt and mutated GPKOW by immunofluorescence analysis demonstrated no apparent differences in localization as they all seemed evenly distributed in the nucleus, but excluded from the nucleoli (Figure 6A). To test if mutation of the PKA-phosphorylation sites in GPKOW would influence GPKOW function *in vivo*, we performed RNA immunoprecipitaion assays with wt (Figure 6B, lanes 1 and 2) and double mutated (Figure 6B, lanes 3 and 4) GPKOW. This resulted in a significant increase (p < 0.01, n = 7) in radiolabelled RNA when the double GPKOW mutant was immunoprecipitated (Figure 6C), suggesting that PKA phosphorylation regulates the ability of GPKOW to bind RNA. Note that over-expression of GPKOW resulted in a more profound difference between the immunoprecipitation control (Figure 6B, lanes 1 and 3) and the GPKOW sample (Figure 6B, lanes 2 and 4) than for the endogenous RNA immunoprecipitation (Figure 4A).

## Discussion

We have characterized the protein GPKOW and demonstrated that it is a nuclear RNA-binding protein which is *in vitro *phosphorylated by and probably associated with the C subunit of PKA. GPKOW contains two PKA phosphorylation sites at S27 and T316, which by mutational analysis were shown to regulate RNA-binding *in vivo *in a PKA-dependent manner. Endogenous as well as expressed wt and mutated GPKOW were all shown to locate to the nucleus, as determined by immunofluorescence microscopy. The mechanism by which GPKOW locates to the nucleus is unknown. However, based on our results, database searches and the literature it probably does not involve PKA-dependent phosphorylation, the G-patch domain or a recognizable nuclear localization signal (NLS). The latter is supported by searches using various publicly available bioinformatics tools including PredictNLS Online, Prosite and PSORT, which all indicated that GPKOW does not contain any known NLS. This was unexpected since the GPKOW homolog, modifier of snc 1 and 2 (MOS2), identified in *Arabidopsis thaliana*, has been shown to be nuclear and contains a NLS [41]. Furthermore, the assumption that nuclear localization of GPKOW do not involve the G-patch domain is supported by recent reports demonstrating that the Zebra fish Telomerase Reverse Transcriptase (zLPTS), which is nuclear, remained nuclear after deletion of its N-terminal G-patch domain [42]. In addition, mutating three of the conserved glycines in the G-patch domain of Tuftelin-Interacting Protein 11 (TFIP11) did not change its nuclear localization [43,44]. In the case of the PKA phosphorylation sites, we show that over-expression of single- or double-mutated GPKOW S27A and GPKOW T316A does not alter their localization.

Based on experiments using RNA immumoprecipitation we concluded that GPKOW binds RNA. However, we can not rule out that the pulled down RNA may be associated with an unknown interaction partner of GPKOW. This needs to be investigated further. We used T1 RNase treatment of the samples prior to immunoprecipitation with anti-GPKOW to obtain suitable sized RNA fragments for the electrophoresis. T1 RNase is an endoribonuclease which digests RNA at G residues. This treatment of cell extracts will therefore result in RNA species of various lengths [25,45]. Hence, a smear of nucleotides when applying anti-GPKOW immunoprecipitation would be expected. However, GPKOW have 3 domains capable of binding nucleic acids that may bind to different but distinct RNAs. That GPKOW binds to specific RNA species finds support in e.g. the results in lane 2 in Figure 4A and lanes 2 and 4 in Figure 6Bwhere faint but distinct RNA bands of different sizes are detected. To what extent these bands represent specific RNAs and hence may imply that GPKOW have affinity for specific RNA sequences remains to be investigated. We can not exclude the possibility that GPKOW also binds DNA in addition to RNA since other G-patch domain containing proteins have been shown to be involved in DNA repair and telomere maintenance [46,42,47] as well as RNA-processing [48,49]. Furthermore, the spliceosomal protein TFIP11, which affects the splicing pattern of the E1A minigene *in vivo *[43], interacts with PRP43 and in that way promotes the release of the lariat-intron [44]. Finally, the KOW motif may also bind both RNA and DNA in that it is frequently found in ribosomal proteins and is proposed to have functions associated with translation [28,50-53]. To this end, a study where two conserved residues were mutated in the KOW motif of UpaY in *Bacteriodes fragilis *revealed loss of function, suggesting a role for the KOW motif in transcriptional termination [54].

The way in which RNA-binding of GPKOW is regulated is not understood but may involve the two sites in GPKOW for PKA-dependent phosphorylation. We postulate these sites since they were both phosphorylated by PKA *in vitro*. In order to determine which site that would be preferred by PKA we performed site-directed mutagenesis. We observed that both single- and double-mutation resulted in a marked decreased phosphorylation, indicating that both S27 and T316 are phosphorylated by PKA. The sequences surrounding GPKOWs PKA phosphorylation sites are RXS^27 ^(arginine-random-serine) and RXT^316 ^(arginine-random-serine). These consensus sequences are estimated to be phosphorylated with low efficiency by PKA [38]. Hence, it may be argued that these sites in GPKOW are only available for *in vitro *phosphorylation and not relevant *in vivo*. However, in support of biological relevance, several physiological substrates for PKA are identified with similar consensus phosphorylation sites. These include the Kir1.1 renal outer medullary K^+ ^channel 1 and 2 (ROMK1/2 at LRKS^200 ^and VRTS^294^) [55-57], the D1 dopamine receptor (DRD1, at KRET^268^) [58], Elongation Factor-2 kinase (eEF2K or calmodulin-dependent protein kinase III at PRRS^499^) [59] and Hormone-sensitive Lipase (HSL, at MRRS^563 ^and PRRS^659^) [60-62]. Together this supports the potential PKA-phosphorylation sites in GPKOW as genuine also *in vivo*.

That PKA may have regulatory effects on RNA-binding *in vivo *finds support in that the SR-protein serine/arginine-rich splicing factor 1 (SRSF1) (Aksaas, Kvissel and Skålhegg, unpublished)[63] and the hnRNP-protein polypyrimidine tract-binding protein (PTB) [40] are both phosphorylated by PKA. Moreover, we have demonstrated that the C subunit of PKA is located to SFCs and regulates alternative splice site selection *in vivo *[7]. Finally, we also recently found that splicing factor arginine/serine-rich 17A (SFRS17A) is an AKAP and may be involved in targeting PKA to SFCs [64]. In the present work we found that GPKOW interacts with Cβ2 as determined by the two-hybrid screen (five of seventeen bait-dependent clones) containing the main binding partner of Cβ2, the R subunit (twelve of seventeen bait dependent clones). Together this supported high specificity of the screen. We also could demonstrate co-immunoprecipitation between GPKOW and Cβ2. We do however note that this interaction appeared fragile as only about 30% of the experiments revealed a clear result. Despite this we considered this interaction likely since the immunoprecipitation conditions were found to be optimal, the immunoprecipitation was not influenced by cAMP and co-immunoprecipitations of GPKOW with Cβ1, Cβ4 or Cα1 were consistently negative. Since the co-immunoprecipitation of GPKOW and Cβ2 was not influenced by cAMP we suggest that the interaction is independent of the R subunit, and hence that GPKOW is not an AKAP. Rather, it should be considered a C-binding protein.

Despite that the biological role of GPKOW is unknown, silencing of its homolog in *C. Elegans *(R11A8.2) by RNAi revealed embryonic lethality [65]. In addition to its importance for embryonic development this result also points to GPKOW as evolutionary conserved, which is suggestive of an important biological role. The latter assumption is also indicated by the importance of G-patch proteins in carcinogenesis [66,67]. MOS2, which contains one G-patch domain and two KOW motifs organized in the same way as GPKOW, is involved in innate immunity via the disease resistance genes in plants [41]. One may speculate that MOS2 may be paralleled to downstream proteins of the Toll-like receptor pathway in eukaryote cells [68]. In immune cells, Toll-like receptors signal through NFκB, which has been shown to bind and activate the PKA C subunit in a cAMP-independent fashion [11]. Cβ2 is an immune cell-specific PKA C subunit [69] and may suggest that Cβ2 is associated with PKA-dependent phosphorylation and regulation of RNA-binding of GPKOW related to immune function. To what extent this is the case remains to be investigated.

## Conclusions

GPKOW is a nuclear protein with three nucleic acid binding domains, one G-patch domain and two KOW-motifs. We have demonstrated that GPKOW binds RNA *in vivo *and that GPKOW associate with Cβ2 of PKA. GPKOWs ability to bind RNA is regulated by PKA-dependent phosphorylation at two sites. This implicates that PKA phosphorylation is a regulatory factor in post-transcriptional RNA processing.

## Competing interests

The authors declare that they have no competing interests.

## Authors' contributions

AKA carried out the experiments and drafted the manuscript. ACVL participated in the plasmid preparations, the immunoprecipitations and the *in vitro *phosphorylation assay. MR participated in the RNA immunoprecipitations. KR participated in the protein purification. AKK participated in the immunofluorescence and contributed to the manuscript drafting. BSS conceived and designed the study, in addition to draft the manuscript. All authors read and approved the final manuscript.

## Supplementary Material

Additional file 1 **Additional Figure 1**. Bait dependency test from a yeast two-hybrid screen, using the PKA C subunit Cβ2 as bait. Click here for file

Additional file 2 **Figure legend to additional Figure 1**. Figure legend describing the bait dependency test from a yeast two-hybrid screen, using the PKA C subunit Cβ2 as bait. Click here for file
