# Supplementary figures and images for: G-patch domain and KOW motifs-containing protein, GPKOW; a nuclear RNA-binding protein regulated by protein kinase A
Source: J Mol Signal. 2011 Aug 31;6:10. doi: 10.1186/1750-2187-6-10 (PMC3179746; doi:10.1186/1750-2187-6-10)

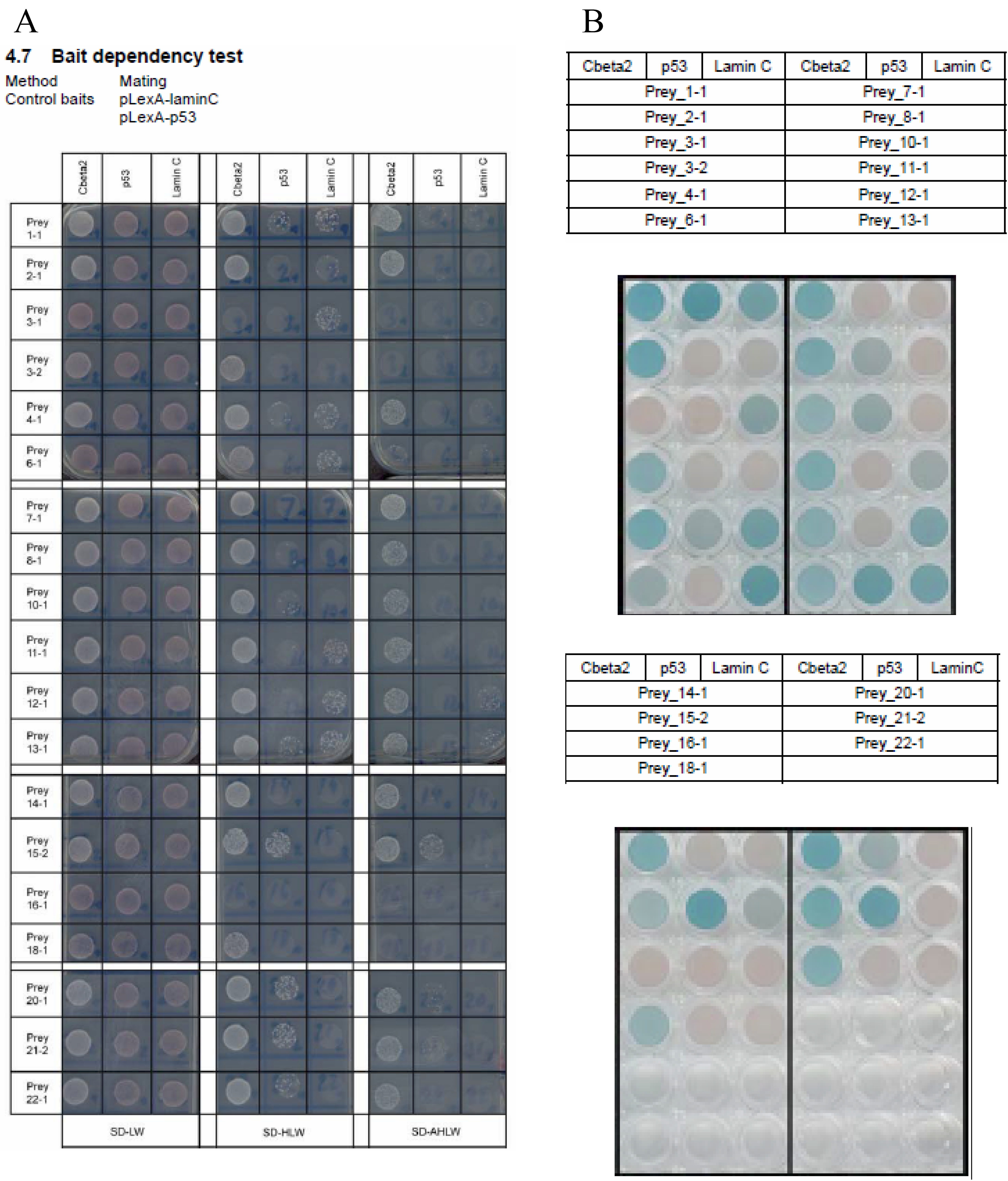

Supplement: Additional file 1 — Additional Figure 1. Bait dependency test from a yeast two-hybrid screen, using the PKA C subunit Cβ2 as bait. [file 1750-2187-6-10-S1.JPEG]
