# Supplementary material for: G-patch domain and KOW motifs-containing protein, GPKOW; a nuclear RNA-binding protein regulated by protein kinase A
Source: J Mol Signal. 2011 Aug 31;6:10. doi: 10.1186/1750-2187-6-10 (PMC3179746; doi:10.1186/1750-2187-6-10)
Supplement: Additional file 2 — Figure legend to additional Figure 1. Figure legend describing the bait dependency test from a yeast two-hybrid screen, using the PKA C subunit Cβ2 as bait. [file 1750-2187-6-10-S2.DOC]

**Additional figure 1. Bait dependency test from a yeast two-hybrid screen, using the PKA C subunit Cβ2 as bait.**

Yeast two-hybrid screen using the PKA Cβ2 as bait was carried out by Dualsystems Biotech. This figure is adapted from their report. Clones 4-1, 8-1, 10-1, 12-1 and 13-1 were identified as GPKOW, while clones 1-1, 2-1, 3-2, 6-1, 7-1, 11-1, 14-1, 15-2, 20-1, 21-2, 22-1 and 18-1 were identified as PKA RIα. **A.** Mating spotted on synthetic defined medium (SD) without leucine (L) and tryptophan (W) (SD-LW the left three lanes) or SD without histidine (H) and LW (SD-HLW the middle three lanes) or SD without adenine (A) and HLW (SD-AHLW the right three lanes). **B.** High throughput β-galactosidase assay with yeast picked from mating plate SD-LW.
